# Cutaneous leishmaniasis in Syria: A review of available data during the war years: 2011–2018

**DOI:** 10.1371/journal.pntd.0007827

**Published:** 2019-12-12

**Authors:** Ghada Muhjazi, Albis Francesco Gabrielli, José Antonio Ruiz-Postigo, Hoda Atta, Mona Osman, Hyam Bashour, Atef Al Tawil, Hania Husseiny, Rasmieh Allahham, Richard Allan

**Affiliations:** 1 Department of Communicable Disease Prevention and Control, WHO/EMRO Regional Office for the Eastern Mediterranean, Cairo, Egypt; 2 Department of Control of Neglected Tropical Diseases, World Health Organization, Geneva, Switzerland; 3 Damascus University, Damascus, Syria; 4 Ministry of Health, Damascus, Syria; 5 Syria WHO Country Office, World Health Organization, Damascus, Syria; 6 The MENTOR Initiative, Crawley, United Kingdom; Universidade do Estado do Rio de Janeiro, BRAZIL

## Abstract

**Background:**

Cutaneous leishmaniasis (CL) has historically been reported from Syria. Since 2011, the country has been affected by a war, which has impacted health and health services. Over the same period, an increase in the number of cases of CL has been reported from several areas across the country and by a number of authors. This study aims to provide the first quantitative evidence of the epidemiological evolution of CL in Syria during the war.

**Materials and methods:**

Data on number of CL cases for the period 2011–2018 were extracted from three different surveillance systems: the Ministry of Health (MoH) routine surveillance system, the MoH/WHO sentinel-syndromic Early Warning Alert and Response System (EWARS), and surveillance data collected by the international nongovernmental organization (NGO) the MENTOR Initiative. Data were cleaned and merged to generate the best possible estimates on number of CL cases; incidence of CL was also calculated based on data on resident population. Data reported from the years preceding the conflict (2007–2010) were also added to the analysis for comparative purposes.

**Results:**

The analysis of data from the three available sources over the period considered indicates that number of reported cases progressively grew from prewar levels to reach a peak in 2015, decreased in 2016, remained stable in 2017, and increased again in 2018. Such a trend was mirrored by changes in incidence of infection. Some governorates, which used to report low numbers of CL cases, started recording higher number of cases after the onset of the war.

**Conclusion:**

The war coincided with a major rise in reported number of CL cases and incidence of infection, although an increasing trend was already appreciable before its onset.

## Introduction

Cutaneous leishmaniasis (CL) has historically been present in Syria, mainly in the western part of the country [1, 2]. Anthroponotic CL due to *Leishmania tropica* is the most common form in the country, but zoonotic CL due to *L*. *major* has also been reported [3, 4]. The overall health situation in Syria has been heavily reshaped by the war (2011–present), due to the deterioration of healthcare infrastructures, challenges in the implementation of control measures, and mass-scale displacement of populations, both within Syria and to other countries (6.2 million internally displaced persons [IDPs] and 5.7 million registered refugees as of March 2019) [5, 6]

The National Leishmaniasis Control Programme (NLCP) was established within the Ministry of Health (MoH) of the Syrian Arab Republic in 1985. During the first 25 years of its life, prevention focused on vector and reservoir control in collaboration with the municipalities and the Ministry of Agriculture. Curative services were implemented by securing laboratory diagnosis and treatment of cases through the available specialized public centers, particularly in the most-affected governorates. Nominal, monthly recording of cases from peripheral health units to central level started in 1985 with the aim of monitoring epidemiological trends and carrying out investigation and response measures when any increase was reported from a given location [7]. Private dermatology clinics were also providing treatment services for the people who could afford them; however, they were not reporting to the MoH thus contributing to the overall underestimation of the number of incident CL cases in Syria [8, 9].

According to unpublished MoH–NLCP data (personal communication, A. Al Tawil), the number of centers specialized in CL and run by the MoH decreased from 267 (124 for diagnosis and treatment and 143 for treatment only) in 2010 to 183 by 2014 as a result of the war, although the number lately increased to 285 by mid-2019 (of which 64 centers are for diagnosis and treatment and 221 for treatment only; the availability of diagnostic facilities is defined by the presence of a medical laboratory at the treatment center), as the MoH designated new facilities for CL case management in areas where the government regained control. In addition, the drainage of healthcare workers able to carry out diagnosis and treatment has further exacerbated the accessibility to, and coverage of, CL services. With regard to prevention, implementation of vector and reservoir control activities has also been significantly and negatively impacted by the war.

Several authors have noted that the onset of the war coincided with a significant increase in number and distribution of CL cases across the country [10–21] and that movements of Syrian refugees led to a general increase in number of CL cases reported from resettlement countries [22–30]. Our analysis focuses on a comprehensive quantification of the evolution of CL epidemiology throughout Syria’s territory, an exercise that, to our knowledge, has not yet been attempted.

This can be explained by the fact that the war has also negatively impacted the CL surveillance and reporting system. While up until the onset of hostilities the MoH routine surveillance system covered the entire national territory, since 2011 the changing frontline between fighting entities has meant that only facilities in areas controlled by the government, and therefore still run by MoH staff, could report to the central units in Damascus.

## Materials and methods

In order to offer the best possible representation of CL epidemiology throughout Syria during the war years, we took into consideration all the available alternative surveillance systems in place during the crisis (2011–2018). Data provided by such surveillance systems were cleaned and merged, based on geographical coverage. Our analysis was extended to data from the years immediately preceding the outbreak of the conflict (2007–2010) for comparative purposes.

The routine surveillance system managed by the MoH was the main source of our data, as it has covered the highest proportion of Syrian territories during the war. The system is a reliable data source and regularly shares information with the World Health Organization (WHO) in compliance with reporting standards. However, limited access to conflict areas resulted in a reporting gap. Reporting failure during the war period was especially high from Idleb (2015–2018), Raqqa (2014–2017), and Deir ez-Zor (2015 and 2017), as well as from the northern part of Aleppo, part of Hama, and part of Hasakeh (2014–2018).

The second data source we used is the Early Warning Alert and Response System (EWARS). EWARS relies on sentinel-based syndromic surveillance and was established by WHO and MoH in September 2012 to capture signals of outbreaks of epidemic-prone diseases, with the aim of triggering a timely response [31]. EWARS was gradually expanded to support—but not replace—the routine surveillance system, which, as mentioned, continues to function in government-controlled areas but was heavily weakened in conflict areas. It should be noted that EWARS data are subject to underreporting for the following reasons: (1) CL is not one of the priority diseases in EWARS system but is, rather, included under the term “other diseases”; (2) EWARS was the only source of data in areas controlled by the Islamic State of Iraq and the Levant (ISIL), such as Raqqa in 2017 and a large portion of Deir ez-Zor in 2015 and 2017, where access to health facilities and data collection were a challenge. Despite this limitation, the MoH used EWARS as the only available source of data from these two “silent governorates” and officially recognized its role.

The third data source, the international nongovernmental organization (NGO) the MENTOR Initiative was the main provider of diagnosis and treatment for CL and consequently the main collector of surveillance data in areas controlled by the Syrian opposition. The MENTOR Initiative was mainly operating in northern Syria during the period of 2014 to 2018. Geographical coverage of services provided by the MENTOR Initiative fluctuated, mirroring the evolution of the security situation on the ground. The MENTOR Initiative relied on a network of over 180 health facilities and mobile clinics and was supported by several international donor organizations—and by WHO—notably through the provision of medicines and long-lasting insecticidal nets (LLINs) to sustain prevention and treatment of CL in areas not accessible by the MoH. Data provided by the MENTOR Initiative (2014–2018) refer to the areas where the NGO was providing services: Raqqa (2014–2016), Idlib (2015–2018), and parts of Aleppo, Hama, and Hasakeh (2014–2018).

The main characteristics of the different data sources (type of surveillance, frequency of data availability, source of data, CL case definition and coverage) are described in Table 1.

**Table 1:** Different data sources and their characteristics.

	Data Source		
	MoH	MENTOR Initiative	EWARS
**Type of Surveillance**	Passive	Passive	Passive
**Availability (frequency)**	Monthly, 1985–present	Monthly, 2014–2018	Weekly, 2012–2018
**Source of data**	Public health facilities	MENTOR health facilities	Public, private and NGOs clinics
**CL defined through / Confirmation**	Syndromic diagnosis /sometimes with lab confirmation	Syndromic diagnosis	Syndromic diagnosis
**Geographical Coverage**	Government-controlled areas (during war period, all governorates except Idleb, Raqqa, Deir ez-Zor, northern Aleppo, some districts in Hama and in Hasakeh)	Opposition-controlled areas (6 districts in North Aleppo, Idleb, 3 districts in Hama, 3 districts in Hasakeh, 3 districts in Raqqa)	All governorates

**Abbreviations**: CL, cutaneous leishmaniasis; EWARS, Early Warning Alert and Response System; MoH, Ministry of Health; NGO, nongovernmental organization

It is worth mentioning that a different surveillance system (Early Warning Alert and Response Network [EWARN]) was also functioning in the northern governorates (in areas controlled by the opposition only). However, EWARN data were not included in our analysis to minimize the risk of duplication of data entries, as the MENTOR Initiative was functioning in almost the same territories and focused specifically on CL.

Data from the three available sources were cleaned, with the aim of avoiding any wrong entry possibly leading to underreporting or overreporting. Data cleaning was both vertical (by ensuring consistency of data reported by a given source at all levels of the health system, through progressive compilation from peripheral to central level) and horizontal, by matching data relevant to the same geographical area (district and subdistrict), to avoid any entry duplication, notably with regard to governorates where more than one surveillance system was operating. As a general rule, primary sources of information were MoH data in areas controlled by the government, and data from the MENTOR Initiative in those controlled by the Syrian opposition, with the exception of Idleb for the year 2014—for which MoH data were used, as health facilities were still reporting to MoH, despite the fact that the military control of the governorate progressively shifted to the opposition. EWARS data were disregarded in areas from where the MoH or the MENTOR Initiative data were available; nevertheless, EWARS was used as a supplementary source in areas, such as those controlled by ISIL, where neither of the mentioned surveillance systems was active.

**Fig 1:**
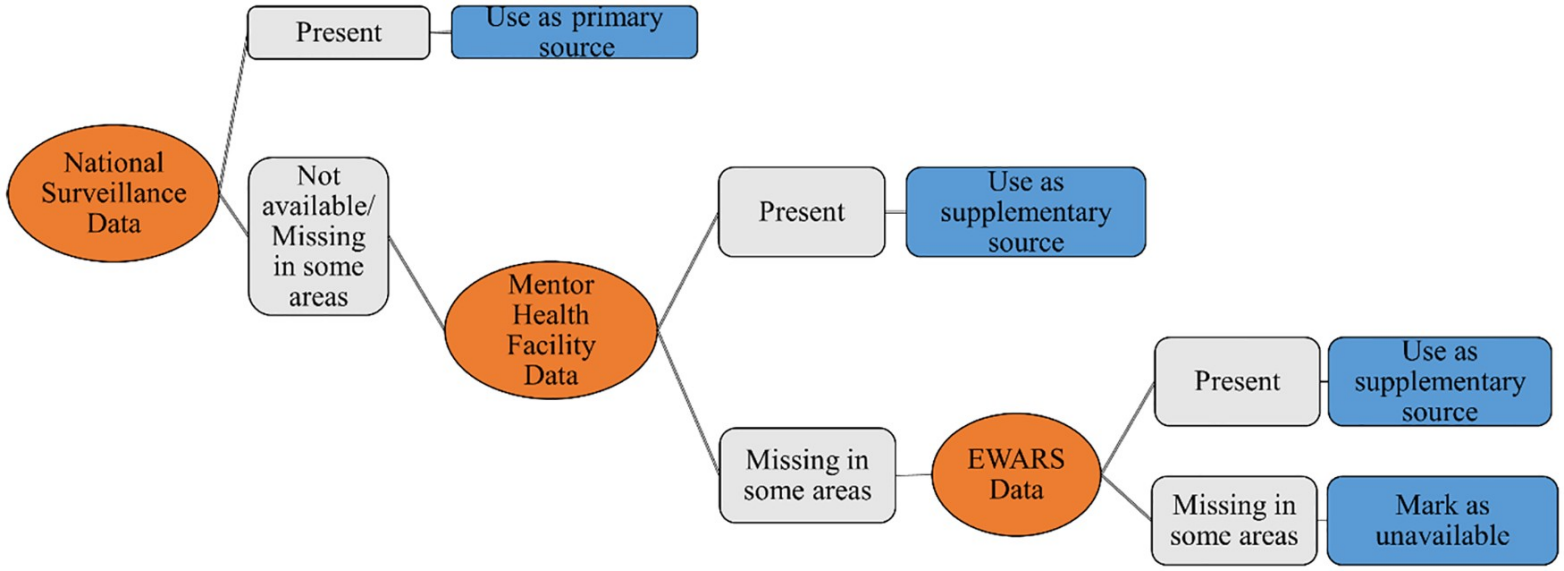
Decision-making process of data inclusion. EWARS, Early Warning Alert and Response System.

Fig 1 illustrates the decision-making process that we followed to complement MoH data with data collected by other surveillance systems. MoH data were used in full, and MENTOR data were used only from areas not covered by MoH. In areas that were neither covered by MoH nor by MENTOR, EWARS data were used.

We focused our analysis on two main indicators: (1) number of new CL cases, nationwide and by governorate, and (2) incidence rate of CL (new cases/10,000 population/year), nationwide. In contrast with the first indicator, the second also reflects changes in the resident population of Syria, which has been steadily declining since 2011; calculations were based on nationwide population data published by the United Nations Department of Economic and Social Affairs (UNDESA), which take into account data on refugee flows compiled by the Office of the United Nations High Commissioner for Refugees (UNHCR) [32]. The nonavailability of reliable population data at governorate or lower level, due to the high fluidity of movements within the country during any given year, prevented us from calculating incidence by governorate and from conducting an in-depth analysis of epidemiological trends at subnational level; for the same reason, we refrained from discussing a causal relationship between fluctuations in number of cases and specific events or risk factors. Nevertheless, we generated maps showing number of CL cases reported in 2010, 2012, 2014, 2016, and 2018, by governorate, with the aim of highlighting the distribution of cases that occurred during the war.

## Results

**Fig 2:**
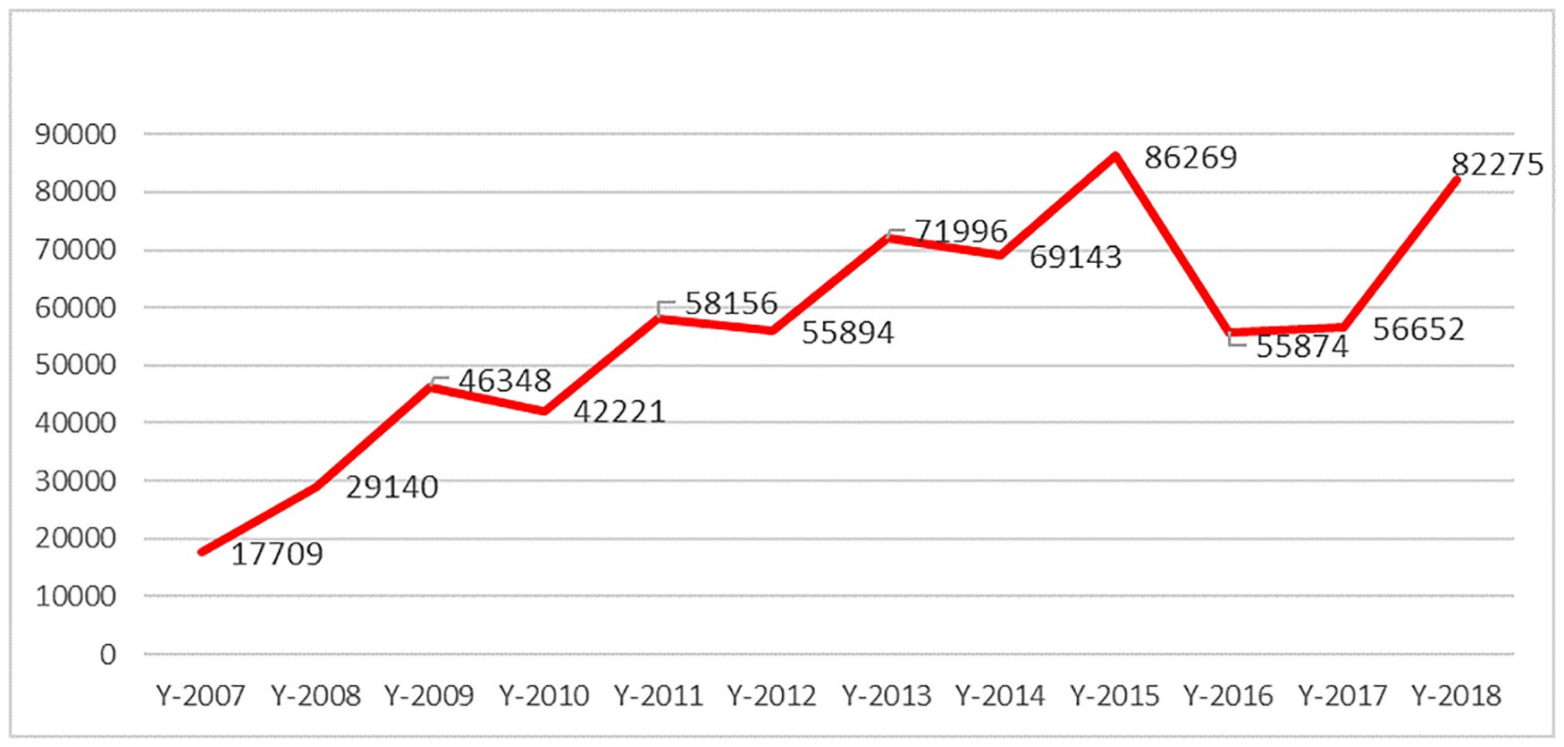
Trend of CL cases (2007–2018) using all sources of data. CL, cutaneous leishmaniasis.

Table 2 shows the number of cases reported by each of the three reporting systems, over the period 2007 to 2018; the last column shows the compounded value resulting from the combination of the three sources, after data cleaning and matching to avoid duplication, as described in the materials and methods section. Fig 2 offers a graphic representation of the same data. Details on how each governorate and each surveillance system contributed to the total are provided in Table 3.

**Table 2:** Number of CL cases reported by different surveillance systems.

Year	MoH	EWARS	MENTOR	All sources (*)
**2007**	17,709			17,709
**2008**	29,140			29,140
**2009**	46,348			46,348
**2010**	42,221			42,221
**2011**	58,156			58,156
**2012**	55,894			55,894
**2013**	71,996			71,996
**2014**	53,876	11,495	27,794	69,143
**2015**	42,390	20,465	43,327	86,269
**2016**	33,486	27,267	22,388	55,874
**2017**	38,864	24,881	15,380	56,652
**2018**	65,795	46,166	20,548	82,275

(*) MoH data + MENTOR (Idleb 2015–2018 + Raqqa 2014–2016) + mix MoH and MENTOR (Aleppo, Hama, and Hasakeh 2014–2018) + EWARS (Raqqa 2017 + Deir ez-Zor 2015 and 2017).

**Abbreviations**: EWARS, Early Warning Alert and Response System; MoH, Ministry of Health

**Table 3:** Number of CL cases disaggregated by year, governorate, and data source.

All sources excluding possible duplication	2007	2008	2009	2010	2011	2012	2013	2014	2015	2016	2017	2018
Damascus	444	766	1,581	1,471	1,320	879	1,026	1,186	772	612	620	1,020
Lattakia	876	849	862	538	510	598	2,467	1,385	475	485	401	662
Tartous	732	676	572	491	641	735	2,832	1,997	1,085	915	868	930
Rural Damascus	351	661	1,007	823	1,123	1,728	2,157	2,542	3,583	2,503	1,386	884
Dara’a	56	67	133	100	77	53	19	7	11	18	48	126
Quneitra	11	3	69	141	14	0	0	2	0	0	22	1
Sweida	28	32	95	57	72	82	85	65	30	46	40	33
Homs	261	455	592	321	201	545	1,489	866	868	866	670	418
Hama	2,302	3,883	5,512	3,780	4,121	3,906	7,616	9,806	16,759	12,233	11,074	12,692
Idleb	1,842	2,219	4,894	4,416	5,324	3,820	12,327	12,699	17,329	10,389	9,230	8,393
Aleppo	10,295	18,603	29,403	23,780	27,712	22,088	22,365	21,743	18,083	11,173	14,715	31,370
Hasakeh	234	394	665	4,727	11,991	14,617	7,524	12,075	22,275	15,559	15,185	11,754
Raqqa	98	186	384	212	415	1,566	1,272	2,183	4,447	106	773	3,502
Deir ez-Zor	179	346	579	1,364	4,635	5,277	10,817	2,587	552	969	1,620	10,490
Total	17,709	29,140	46,348	42,221	58,156	55,894	71,996	69,143	86,269	55,874	56,652	82,275

Color keys for data sources: White: MoH; Green: The MENTOR Initiative; Yellow: EWARS; Orange: MoH + MENTOR.

**Abbreviations**: CL, cutaneous leishmaniasis; MoH, Ministry of Health

Despite the limitations inherent to the context in which they were collected, if we consider the baseline year as 2010—that is, the year prior to the war when the surveillance system was reportedly functioning well throughout the national territory—data show a considerable increase in the number of CL cases between 2010 and 2018, although fluctuating from year to year and adding to an increasing trend evident since 2007.

The yearly evolution of the number of CL cases in Syria is reflected by the nationwide trend of incidence rate per population of 10,000. Between 2010, the year prior to the onset of the war, and 2018, the incidence rate more than doubled, from 20.08 to 44.99 (Table 4).

**Table 4:** Increase in CL incidence rate per 10,000 population.

Year	Number of cases from all sources*	Population	Incidence rate per 10,000
2007	17,709	19,632,806	9.020106
2008	29,140	20,325,443	14.33671
2009	46,348	20,824,893	22.25606
2010	42,221	21,018,834	20.08722
2011: Onset of war	58,156	20,863,993	27.87386
2012	55,894	20,420,701	27.37124
2013	71,996	19,809,141	36.34484
2014	69,143	19,203,090	36.00618
2015	86,269	18,734,987	46.047
2016	55,874	18,430,453	30.31612
2017	56,652	18,269,868	31.00843
2018	82,275	18,284,407	44.99736
Increase in the incidence rate between 2010 to 2018			**24.91036**

*MoH is the only source of data for the period 2007–2013

**Abbreviations**: CL, cutaneous leishmaniasis; MoH, Ministry of Health

Distribution of CL cases within the country also evolved during the war. Some governorates, which used to report few numbers of cases, started recording higher levels of incidence. Striking examples include Hasakeh in the north-eastern part of the country, where, in 2010, the number of cases reported was 4,727. This figure was almost five times higher in 2015 (22,275). Raqqa reported just 212 cases in 2010, but annual case reports increased sharply, reaching a total of 4,447 annual cases by 2015, a 22-fold increase.

**Fig 3:**
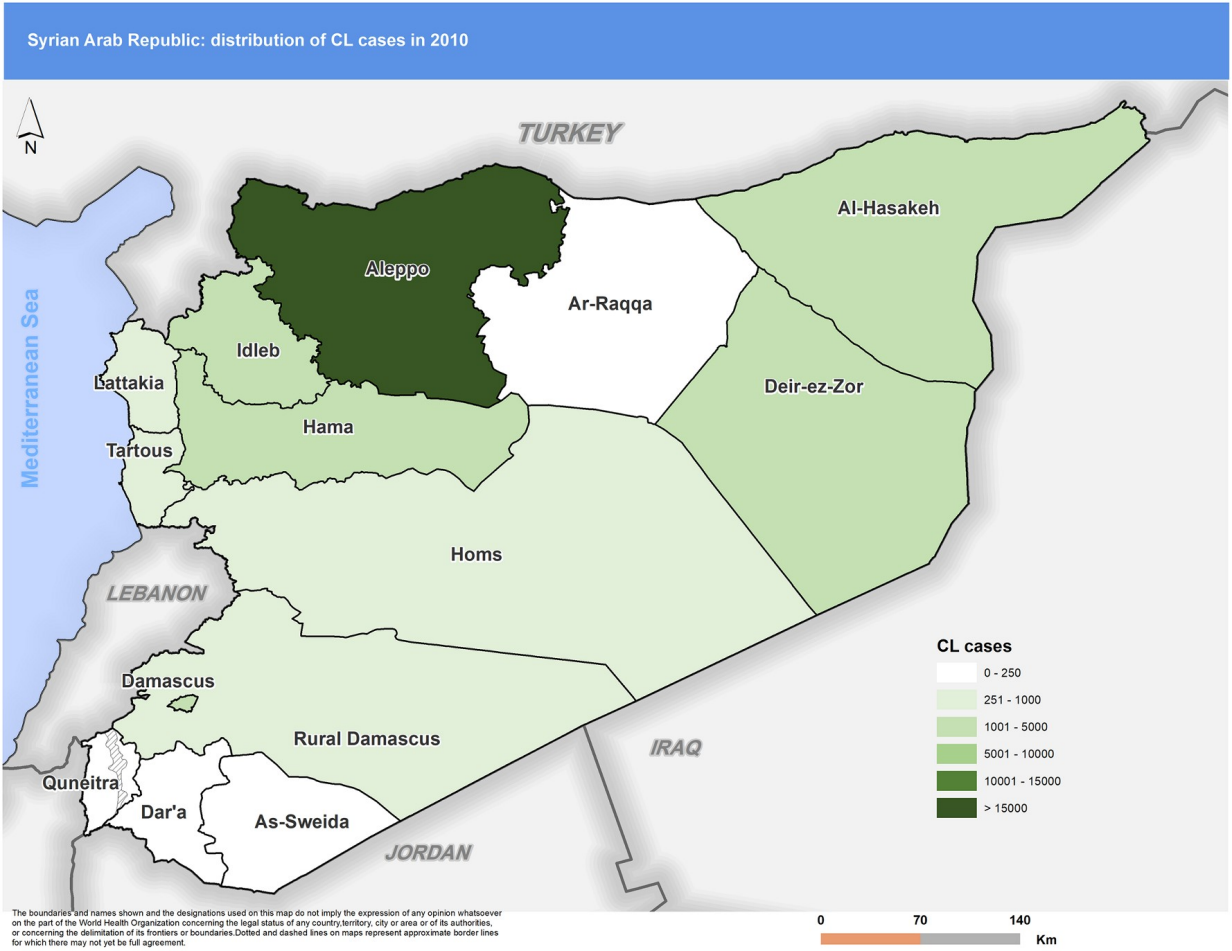
Distribution of CL cases by governorate, 2010. CL, cutaneous leishmaniasis.

**Fig 4:**
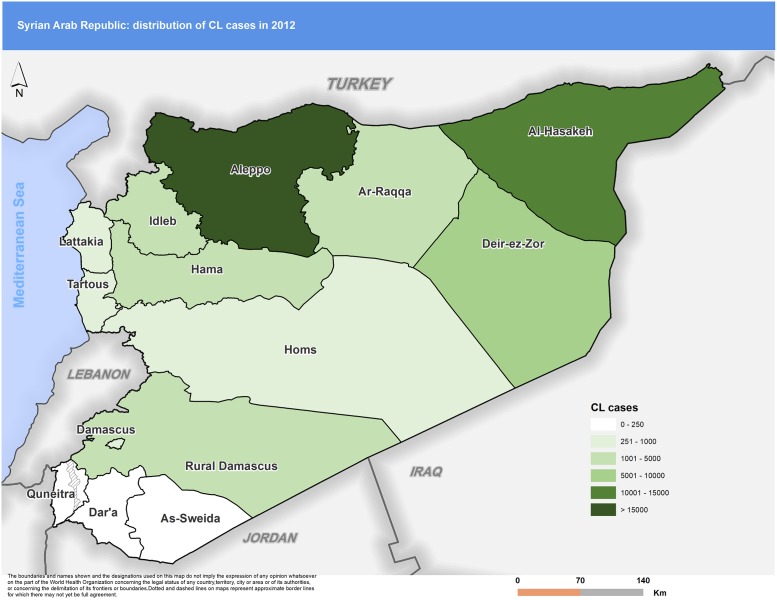
Distribution of CL cases by governorate, 2012. CL, cutaneous leishmaniasis.

**Fig 5:**
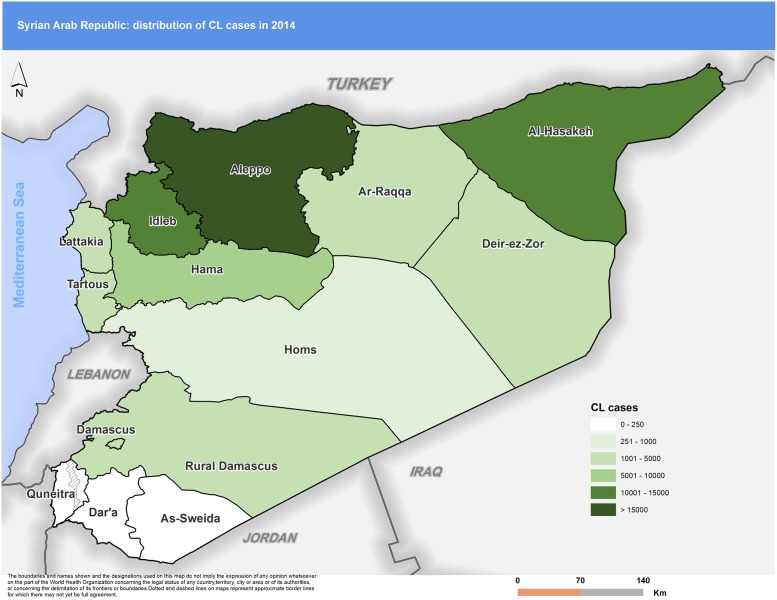
Distribution of CL cases by governorate, 2014. CL, cutaneous leishmaniasis.

**Fig 6:**
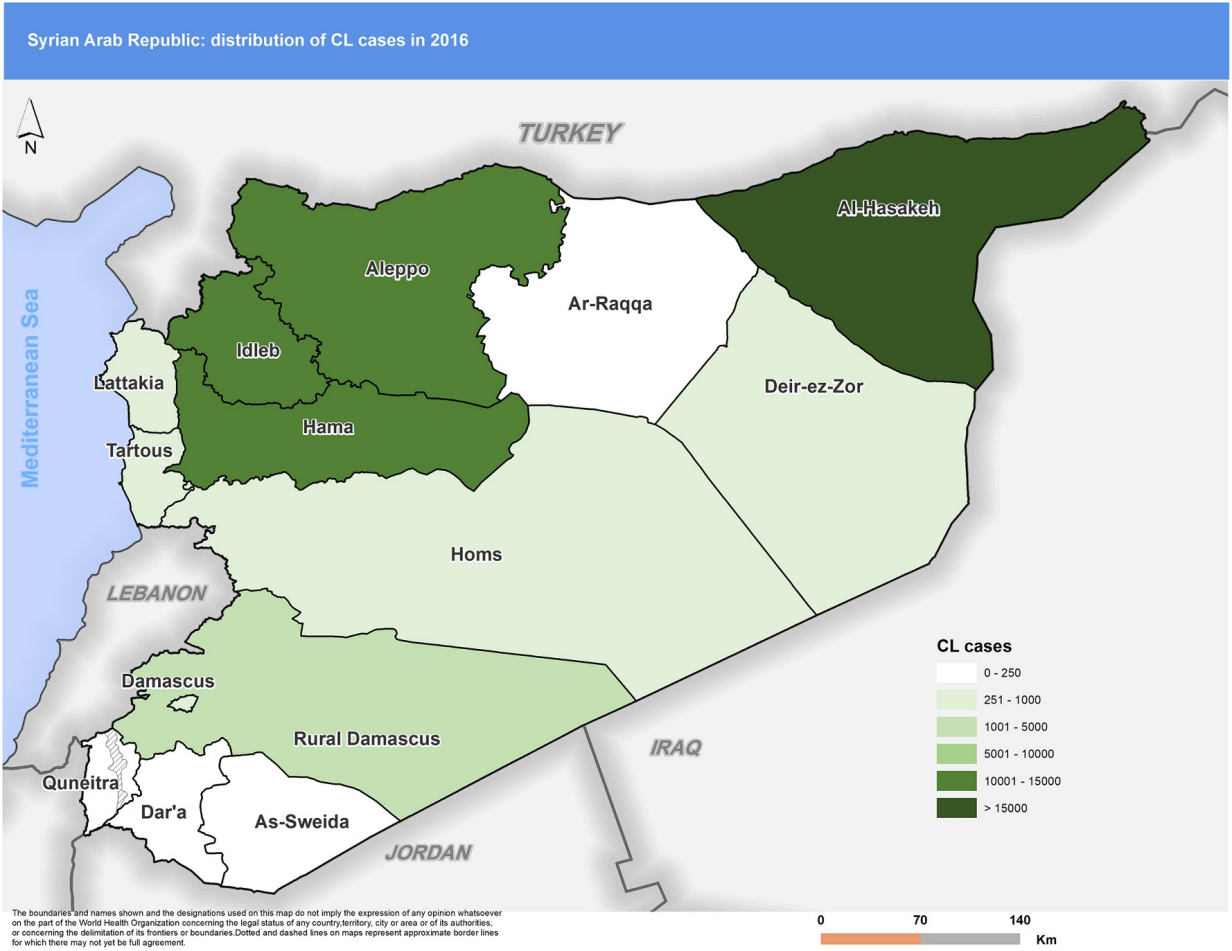
Distribution of CL cases by governorate, 2016. CL, cutaneous leishmaniasis.

**Fig 7:**
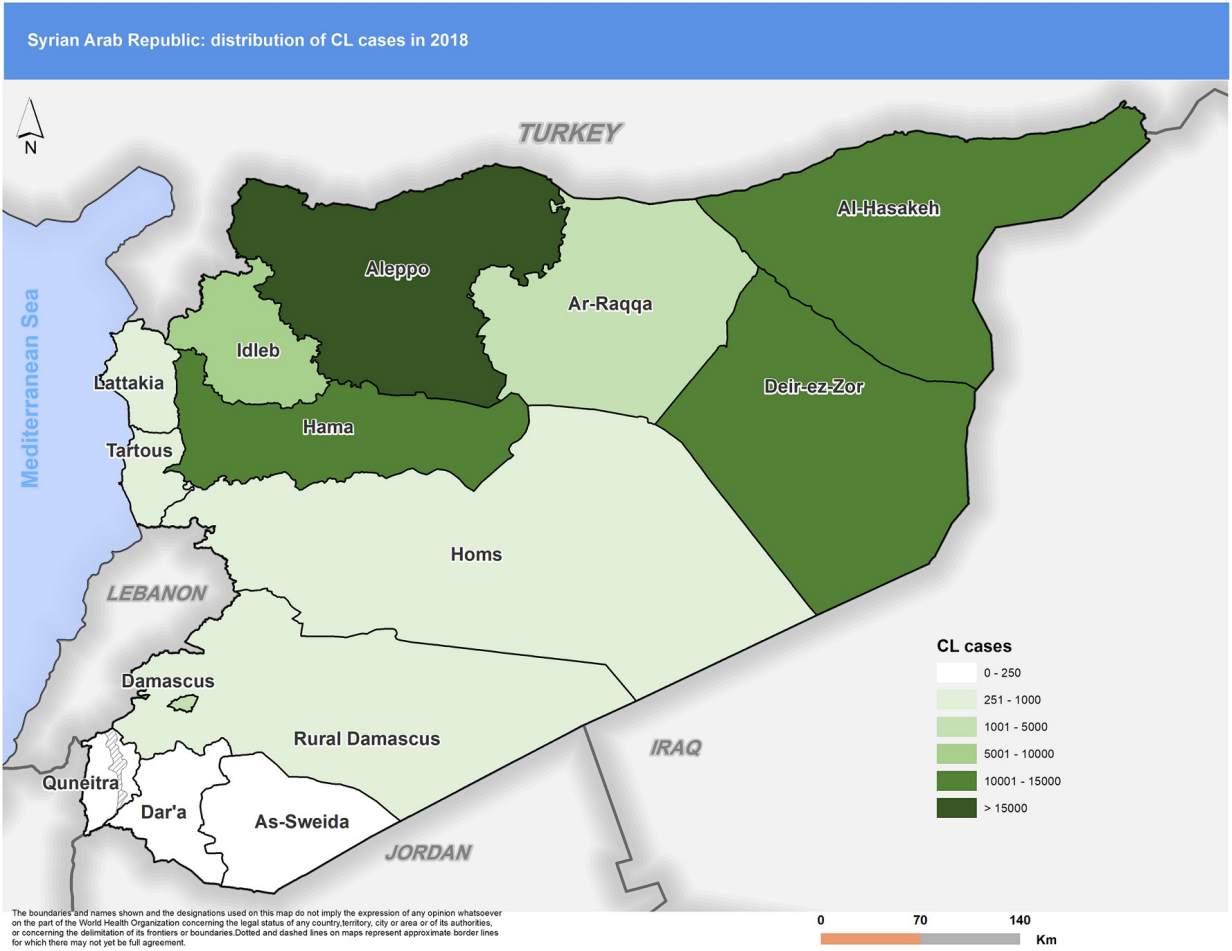
Distribution of CL cases by governorate, 2018. CL, cutaneous leishmaniasis.

Figs 3 to 7 highlight the changes occurring in the distribution of cases between 2010 and 2018. In 2010 Aleppo used to report the highest number of cases. Eight years later, Aleppo is still reporting the highest number of cases, but dispersion of cases across the country is more evident.

## Discussion

The relationship between war and emergence of infectious diseases, including leishmaniasis, is well known throughout the history of medicine and has been the subject of extensive literature research [33–36]. Evidence attributes responsibility to a wide range of risk factors including population displacement and precarious resettling, environmental conditions, breakdown of control measures, collapse of the health system and decrease in health workforce, and inadequate surveillance and detection of cases, as well as impeded access to health facilities and to affected populations [37].

Our study proceeds in the above steps, and, following a series of reports on increased incidence of CL in Syria, it examines the evolution of the epidemiology of this disease during the years of the war, when most of the mentioned risk factors were fiercely present [10]. Notably, through a comprehensive and consistent compilation and analysis of the different operating surveillance systems, it provides the first quantitative estimates on number of incident CL cases by governorate and on incidence rate of CL for the whole of Syria, thus allowing a comparison with prewar years.

The main findings of our data analysis exercise suggest that the progressive increase in the number of CL cases was already evident between 2007 and 2010. The surge observed since the onset of the war, which led to doubling the number of cases between 2010 and 2018, therefore adds to a progressively increasing trend that was already in place and that fits into a pattern observed in other Middle Eastern countries, whereby cyclic increases and decreases in the number of cases occurs every 7 to 10 years [38–39]; with regard to the Aleppo governorate, for example, a progressive increase in incidence of CL had been documented since the mid-1980s [2, 40], in spite of extensive vector control efforts [41].

The significant increase in CL incidence between 2010 and 2011 cannot be attributed to the conflict, due to its still limited impact on infrastructures and populations; in our view it is largely due to the ongoing, increasing epidemiological trend. The slight decrease in the reported number of cases registered in 2012 in comparison with 2011 finds its probable cause in the breakdown of surveillance and failed reporting occurring during the first year of the war, as both the MoH health and surveillance networks were affected and no major alternative health providers or surveillance systems were yet in place; notably, EWARS was established in 2012, but the number of sentinel sites was still very small, and none of them were yet reporting disaggregated data on leishmaniasis. The same consideration applies to 2013, a year that nevertheless sees a remarkable increase in number of cases, in spite of the reporting gap caused by the decreasing coverage of MoH facilities and the absence of an alternative surveillance system (still limited functionality of EWARS and the MENTOR Initiative was not yet operational).

In 2014, both EWARS and the MENTOR Initiative start contributing to CL data reporting; in our opinion, data from this year onwards become increasingly representative of the true epidemiological situation of CL in Syria, as surveillance is no longer hampered by major coverage gaps. While we refrain from discussing a causal relationship between specific events and fluctuations in the number of cases, we note that the large numbers reported in 2014 and 2015 parallel the destruction of infrastructure in the traditional disease epicenters of Aleppo and Idleb and the resulting mass population movement, as well as the onset of war in Hasakeh and Lattakia.

The decreasing trend in detected number of cases in 2016 to 2017 can possibly be attributed, at least in part (and in addition to progressing herd immunity [42]), to increasing attention to CL by national and international health actors, which resulted in intensified implementation of preventive measures, such as distribution of insecticide-treated nets and focal vector control, increased availability of medicines, and improved access to health services [43–47] (as mentioned, the MENTOR Initiative starts operating in northern Syria in 2014). The post-2015 decrease is confirmed by the reduction in incidence, which provides a more accurate insight as it reflects changes in the resident population. Nevertheless, in 2018, both number of cases and incidence rate increased again, indicating that the epidemiological situation is still unstable and susceptible to change. Aleppo and Deir ez-Zor governorates are the main contributors to the worsening registered in 2018. The progressive political stabilization of such two governorates could be one of the factors leading to an increased reporting, due to a backlog of previously unattended persons who had better access to health services during 2018.

In addition to increased or decreased access to health facilities, the changing distribution of cases among governorates may also reflect evolutions in the transmission patterns in the geographical area under consideration, possibly produced by the varying exposure to risk factors for infection or by the targeted implementation (or discontinuation) of vector control measures. Mass population movements also appear to have played a role in reshaping the epidemiological pattern of CL within the country. Observed decreases in number of cases may be attributed to movement out of endemic areas into less-affected parts of the country, into areas with limited access to health services, or into neighboring countries; conversely, observed increase in some governorates could also reflect movements of infected people during the incubation period, as the lesions would appear in the new settlement area and would be reported as local cases. For example, population movements from highly-endemic areas in north-western Syria affected by war (e.g., Aleppo) to safer governorates in the south, north-east, and west may explain shifts in reported number of cases, as recorded in Lattakia [20].

It should be noted, however, that the attribution of a case to a specific geographical location is not always a straightforward process. Although MoH instructions prescribe that cases should always be attributed to the area of likely infection of the patient and not to the place of diagnosis, the implementation of such policy has somewhat relaxed over the war period as a result of frequent population displacement, which undermined the rationale itself of the policy. As a consequence, for example, health centers supported by the MENTOR Initiative recorded case location as the place in which displaced people had settled. We acknowledge that a comprehensive understanding of the evolution of CL epidemiology at subnational level, as required by the focal nature of CL, is hampered by the above consideration, as well as by the impossibility to calculate the incidence rate by governorate or district, due to the frequency and extension of population movement and the marked fluidity of the situation.

Additional limitations of our analysis are inherent to the risk of overreporting and underreporting of CL cases. Risk of overreporting is linked to the fact that the same patients may be reregistered as “new cases” if they contact different health centers before the completion of the course of treatment or if they experience a relapse. Based on field assessments conducted in a sample of governorates, however, we reached the conclusion that duplication of reporting is not so relevant because the three sources of data operate in distinct areas (at governorate or district level) and because standard operating procedures are in place to minimize the risk of double registration of the same patient [48]. Underreporting is inherent to a disease such as CL, whose lesions can be small-sized, painless, and self-healing, thus possibly discouraging health-seeking behavior; as such, underreporting is found in all countries where CL is transmitted. Nevertheless, in Syria, risk of underreporting is increased by the fact that during some phases of the war, in some governorates or districts, availability of medicines and access to health facilities were severely limited. It is therefore likely that some CL cases could not be attended to and as such were neither registered nor reported. Such challenges were substantial, especially in areas controlled by ISIL, as well as in areas experiencing open fighting. The considerations made on EWARS in the Materials and Methods section (i.e., the inclusion of CL among the “other diseases” section of the form) also entail a risk of underreporting. Finally, it should be noted that the private sector does not report cases to the MoH. However, private clinics are limited mainly to urban areas and are used by the wealthiest sectors of the population. In addition, only few of them have continued to operate in war areas. It is therefore our conviction that underreporting due to attendance of private facilities is not substantial.

## Conclusion

The war in Syria has been associated with an increase in risk factors for CL, such as reduced implementation of preventive measures, impaired access to diagnosis and treatment, destruction of infrastructures, population movements, and worsening living conditions, including overcrowding. During the same time period, an increase in number of cases of CL was reported from multiple sources, although never exhaustively quantified. In spite of the inherent limitations of our analysis, we believe that this study offers a reliable, comprehensive, and systematic overview of the evolution of CL epidemiology in Syria since the onset of the war in 2011. Looking towards the future, it is important to ensure that surveillance, prevention, diagnosis, and treatment of CL are increasingly strengthened so as to allow for a proper management of the associated burden of disease. It is clear, however, that only a resolution of the war may allow for proper implementation of such measures. This is indeed our hope.
